# 2-Benzyl­sulfanyl-4-pentyl-6-(phenyl­sulfan­yl)pyrimidine-5-carbonitrile

**DOI:** 10.1107/S1600536811044746

**Published:** 2011-10-29

**Authors:** Ali A. El-Emam, Omar A. Al-Deeb, Abdulghafoor A. Al-Turkistani, Seik Weng Ng, Edward R. T. Tiekink

**Affiliations:** a Department of Pharmaceutical Chemistry, College of Pharmacy, King Saud University, Riyadh, Saudi Arabia; b Department of Chemistry, University of Malaya, 50603 Kuala Lumpur, Malaysia; c Chemistry Department, Faculty of Science, King Abdulaziz University, PO Box 80203 Jeddah, Saudi Arabia

## Abstract

In the title pyrimidine derivative, C_23_H_23_N_3_S_2_, the phenyl­sulfanyl and benzyl­sulfanyl benzene rings are orientated away from the carbonitrile group and are twisted out of the plane of the central ring with dihedral angles of 77.66 (6) and 64.73 (5)°, respectively. The *n*-pentyl group has an extended *trans* conformation. In the crystal, supra­molecular layers in the *ab* plane are sustained by C—H⋯π and π–π inter­actions [pyrimidine–phenyl­sulfanyl centroid–centroid distance = 3.8087 (7) Å].

## Related literature

For the chemotherapeutic activity of pyrimidine derivatives, see: Al-Safarjalani *et al.* (2005 ▶); Pauwels (2004 ▶); Hawser *et al.* (2006 ▶), Al-Omar *et al.* (2010 ▶); Al-Abdullah *et al.* (2011 ▶). For a related pyrimidine structure, see: Nasir *et al.* (2010 ▶).
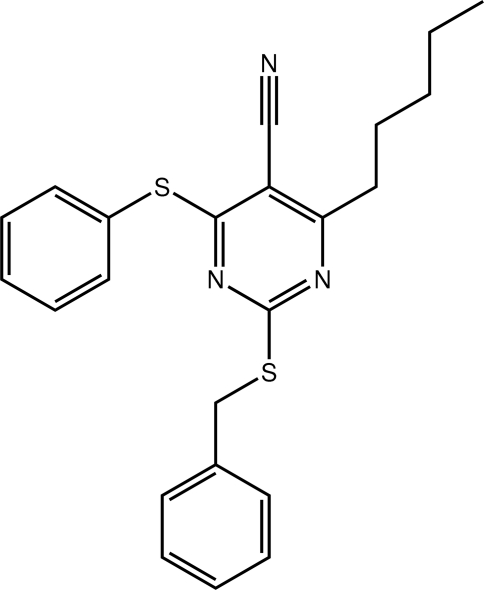


## Experimental

### 

#### Crystal data

C_23_H_23_N_3_S_2_
*M* *_r_* = 405.56
Monoclinic, 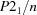

*a* = 9.0093 (1) Å
*b* = 8.2137 (1) Å
*c* = 28.6398 (3) Å
β = 98.427 (1)°
*V* = 2096.45 (4) Å^3^
*Z* = 4
Cu *K*α radiation
μ = 2.39 mm^−1^
*T* = 100 K
0.25 × 0.25 × 0.15 mm

#### Data collection

Agilent SuperNova Dual diffractometer with an Atlas detector
Absorption correction: multi-scan (*CrysAlis PRO*: Agilent, 2010 ▶) *T*_min_ = 0.586, *T*_max_ = 0.715
8836 measured reflections
4307 independent reflections
4029 reflections with *I* > 2σ(*I*)
*R*_int_ = 0.017

#### Refinement

*R*[*F* ^2^ > 2σ(*F* ^2^)] = 0.030
*wR*(*F* ^2^) = 0.084
*S* = 1.02
4307 reflections
253 parameters
H-atom parameters constrained
Δρ_max_ = 0.26 e Å^−3^
Δρ_min_ = −0.26 e Å^−3^

### 

Data collection: *CrysAlis PRO* (Agilent, 2010 ▶); cell refinement: *CrysAlis PRO*; data reduction: *CrysAlis PRO*; program(s) used to solve structure: *SHELXS97* (Sheldrick, 2008 ▶); program(s) used to refine structure: *SHELXL97* (Sheldrick, 2008 ▶); molecular graphics: *ORTEP-3* (Farrugia, 1997 ▶) and *DIAMOND* (Brandenburg, 2006 ▶); software used to prepare material for publication: *publCIF* (Westrip, 2010 ▶).

## Supplementary Material

Crystal structure: contains datablock(s) global, I. DOI: 10.1107/S1600536811044746/hg5126sup1.cif

Structure factors: contains datablock(s) I. DOI: 10.1107/S1600536811044746/hg5126Isup2.hkl

Supplementary material file. DOI: 10.1107/S1600536811044746/hg5126Isup3.cml

Additional supplementary materials:  crystallographic information; 3D view; checkCIF report

## Figures and Tables

**Table 1:** Hydrogen-bond geometry (Å, °) *Cg*1 is the centroid of the C6–C11 ring.

*D*—H⋯*A*	*D*—H	H⋯*A*	*D*⋯*A*	*D*—H⋯*A*
C21—H21b⋯*Cg*1^i^	0.99	3.00	3.8443 (14)	148

Symmetry code: (i) 
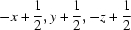
.

**Fig. 1.**
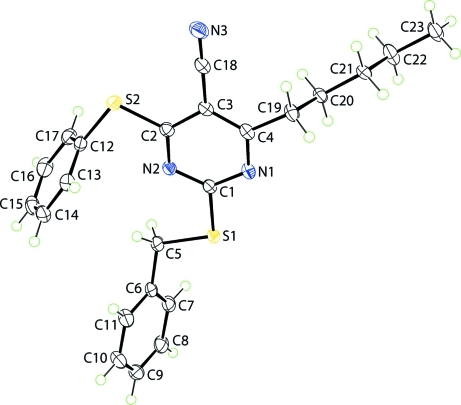
The molecular structure of (I) showing the atom-labelling scheme and displacement ellipsoids at the 50% probability level.

**Fig. 2.**
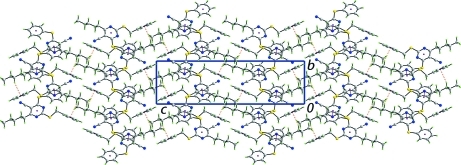
Unit-cell contents for (I) shown in projection down the a axis. The C—H···π and π–π interactions are shown as orange and purple dashed lines, respectively.

## supplementary crystallographic information

### Comment

The chemotherapeutic efficacy of pyrimidine derivatives is related to their ability to inhibit vital enzymes responsible for DNA biosynthesis. A large array of pyrimidine non-nucleoside derivatives possess various chemotherapeutic properties. These properties include anti-cancer (Al-Safarjalani *et al.*, 2005), anti-viral (Pauwels, 2004), anti-bacterial (Hawser *et al.*, 2006; Al-Abdullah *et al.*, 2011). In continuation to our interest in the chemical and pharmacological properties of pyrimidine-5-carbonitrile derivatives (Al-Omar *et al.*, 2010; Al-Abdullah *et al.*, 2011), we synthesized the title compound, (I), as a potential chemotherapeutic agent, and as part of on-going structural studies of pyrimidine derivatives (Nasir *et al.*, 2010), the crystal structure determination is reported herein.

The molecule of (I), Fig. 1, is a tetra-substituted pyrimidine derivative. With reference to the pyrimidine ring, the *S*-benzene and benzyl-benzene rings are each twisted out of the plane as indicated in the respective dihedral angles of 77.66 (6) and 64.73 (5)°. The dihedral angle between the benzene rings is 51.74 (6)°, indicating a non-parallel orientation, and they are directed to the same side of the molecule, *i.e*. away from the carbonitrile group. The *n*-pentyl group has an extended *trans*-conformation: the range of torsion angles = 174.92 (10) to -179.41 (12)°.

Weak C—H···π, Table 1, and π–π interactions feature in the crystal packing. The π–π interactions occur between the pyrimidine and *S*-benzene ring with the separation between the ring centroids being 3.8087 (7) Å [angle between rings = 14.45 (6)° for symmetry operation 3/2 - *x*, 1/2 + *y*, 1/2 - *z*]. The C—H···π interaction involves a methylene-H atom interacting with the benzyl-benzene ring. The interactions lead to supramolecular layers that inter-digitate along the *c* axis. Globally, the crystal structure comprises alternating pyrimidine-rich and aromatic regions stacking along the *c* direction.

### Experimental

To a solution of 2-(benzylthio)-4-chloro-6-(*n*-pentyl)pyrimidine-5-carbonitrile (665 mg, 2.0 mmol) in dry pyridine (3 ml) was added thiophenol (220 mg, 2.0 mmol). The mixture was heated for 6 h. On cooling, the solvent was distilled off *in vacuo*, and water (5 ml) was added to the residue. The precipitate was filtered, washed with cold water, dried and crystallized from ethanol to yield 625 mg (77%) of the title compound as colourless crystals, *M*.pt. 373–375 K. ^1^H NMR (DMSO-d_6_): δ 0.86 (t, 3H, CH_3_, J = 7.0 Hz), 1.30–1.33 (m, 4H, CH_2_CH_2_CH_3_), 1.66–1.69 (m, 2H, CH_2_—CH_2_CH_2_CH_3_), 2.77 (t, 2H, CH_2_—CH_2_CH_2_CH_2_CH_3_, J = 7.0 Hz), 3.99 (s, 2H, CH_2_S), 6.99–7.0 (m, 2H, Ar—H), 7.15–7.22 (m, 3H, Ar—H), 7.50–7.52 (m, 3H, Ar—H), 7.66–7.68 (m, 2H, Ar—H). ^13^C NMR: 13.70 (CH_3_), 21.72 (CH_2_CH_3_), 26.93 (CH_2_CH_2_CH_3_), 30.62 (CH_2_CH_2_CH_2_CH_3_), 33.97 (CH_2_CH_2_CH_2_CH_2_CH_3_), 35.64 (CH_2_S), 98.94 (pym. ring), 114.22 (CN), 125.17, 127.12, 128.30, 128.63, 129.56, 130.47, 135.81, 137.0 (Ar—C), 171.98, 172.71, 172.85 (pym. ring).

### Refinement

Carbon-bound H-atoms were placed in calculated positions [C—H 0.95 to 0.99 Å, *U*_iso_(H) 1.2 to 1.5*U*_eq_(C)] and were included in the refinement in the riding model approximation. One reflection, *i.e*. (002), was omitted owing to poor agreement.

### Crystal data

**Table:**

C_23_H_23_N_3_S_2_	*F*(000) = 856
*M**_r_* = 405.56	*D*_x_ = 1.285 Mg m^−^^3^
Monoclinic, *P*2_1_/*n*	Cu *K*α radiation, λ = 1.54184 Å
Hall symbol: -P 2yn	Cell parameters from 5846 reflections
*a* = 9.0093 (1) Å	θ = 3.1–76.4°
*b* = 8.2137 (1) Å	µ = 2.39 mm^−^^1^
*c* = 28.6398 (3) Å	*T* = 100 K
β = 98.427 (1)°	Block, colourless
*V* = 2096.45 (4) Å^3^	0.25 × 0.25 × 0.15 mm
*Z* = 4	

### Data collection

**Table:**

Agilent SuperNova Dual diffractometer with an Atlas detector	4307 independent reflections
Radiation source: SuperNova (Cu) X-ray Source	4029 reflections with *I* > 2σ(*I*)
Mirror	*R*_int_ = 0.017
Detector resolution: 10.4041 pixels mm^-1^	θ_max_ = 76.6°, θ_min_ = 5.0°
ω scan	*h* = −11→9
Absorption correction: multi-scan (*CrysAlis PRO*: Agilent, 2010)	*k* = −10→10
*T*_min_ = 0.586, *T*_max_ = 0.715	*l* = −35→33
8836 measured reflections	

### Refinement

**Table:**

Refinement on *F*^2^	Primary atom site location: structure-invariant direct methods
Least-squares matrix: full	Secondary atom site location: difference Fourier map
*R*[*F*^2^ > 2σ(*F*^2^)] = 0.030	Hydrogen site location: inferred from neighbouring sites
*wR*(*F*^2^) = 0.084	H-atom parameters constrained
*S* = 1.02	*w* = 1/[σ^2^(*F*_o_^2^) + (0.0513*P*)^2^ + 0.5251*P*] where *P* = (*F*_o_^2^ + 2*F*_c_^2^)/3
4307 reflections	(Δ/σ)_max_ = 0.001
253 parameters	Δρ_max_ = 0.26 e Å^−^^3^
0 restraints	Δρ_min_ = −0.26 e Å^−^^3^

### Fractional atomic coordinates and isotropic or equivalent isotropic displacement parameters (Å^2^)

**Table:**

	*x*	*y*	*z*	*U*_iso_*/*U*_eq_	
S1	0.30604 (3)	0.80793 (4)	0.220499 (10)	0.02127 (9)	
S2	0.67551 (3)	0.34287 (4)	0.296075 (10)	0.02388 (9)	
N1	0.40792 (11)	0.79888 (13)	0.31052 (3)	0.0209 (2)	
N2	0.48869 (10)	0.58292 (12)	0.26357 (3)	0.0190 (2)	
N3	0.73908 (13)	0.46797 (16)	0.41882 (4)	0.0307 (3)	
C1	0.41211 (12)	0.71875 (14)	0.26995 (4)	0.0182 (2)	
C2	0.56944 (12)	0.52008 (14)	0.30172 (4)	0.0189 (2)	
C3	0.57250 (12)	0.59182 (15)	0.34636 (4)	0.0196 (2)	
C4	0.48934 (12)	0.73471 (15)	0.34910 (4)	0.0203 (2)	
C5	0.33729 (16)	0.66417 (16)	0.17420 (4)	0.0272 (3)	
H5A	0.4438	0.6301	0.1787	0.033*	
H5B	0.2746	0.5660	0.1760	0.033*	
C6	0.29747 (14)	0.74341 (15)	0.12657 (4)	0.0217 (2)	
C7	0.14885 (14)	0.77277 (16)	0.10733 (4)	0.0244 (3)	
H7	0.0705	0.7464	0.1249	0.029*	
C8	0.11432 (16)	0.84024 (17)	0.06264 (5)	0.0292 (3)	
H8	0.0126	0.8595	0.0498	0.035*	
C9	0.22769 (17)	0.87966 (17)	0.03667 (5)	0.0325 (3)	
H9	0.2038	0.9244	0.0059	0.039*	
C10	0.37576 (17)	0.85339 (18)	0.05583 (5)	0.0335 (3)	
H10	0.4538	0.8814	0.0383	0.040*	
C11	0.41074 (14)	0.78623 (17)	0.10058 (5)	0.0283 (3)	
H11	0.5128	0.7693	0.1136	0.034*	
C12	0.63604 (13)	0.31303 (15)	0.23406 (4)	0.0215 (2)	
C13	0.70954 (14)	0.40745 (16)	0.20428 (5)	0.0261 (3)	
H13	0.7836	0.4840	0.2170	0.031*	
C14	0.67381 (15)	0.38880 (19)	0.15583 (5)	0.0319 (3)	
H14	0.7212	0.4550	0.1352	0.038*	
C15	0.56860 (16)	0.2732 (2)	0.13749 (5)	0.0338 (3)	
H15	0.5447	0.2600	0.1043	0.041*	
C16	0.49858 (15)	0.17724 (18)	0.16746 (5)	0.0320 (3)	
H16	0.4283	0.0968	0.1548	0.038*	
C17	0.53048 (14)	0.19798 (16)	0.21598 (5)	0.0255 (3)	
H17	0.4806	0.1341	0.2366	0.031*	
C18	0.66434 (13)	0.52388 (15)	0.38680 (4)	0.0226 (2)	
C19	0.48584 (13)	0.82239 (16)	0.39484 (4)	0.0235 (3)	
H19A	0.5041	0.9398	0.3903	0.028*	
H19B	0.5677	0.7807	0.4187	0.028*	
C20	0.33583 (13)	0.80131 (15)	0.41337 (4)	0.0215 (2)	
H20A	0.2549	0.8522	0.3910	0.026*	
H20B	0.3130	0.6838	0.4153	0.026*	
C21	0.33978 (13)	0.87849 (16)	0.46189 (4)	0.0224 (2)	
H21A	0.4233	0.8297	0.4837	0.027*	
H21B	0.3608	0.9962	0.4595	0.027*	
C22	0.19546 (16)	0.85754 (19)	0.48265 (5)	0.0326 (3)	
H22A	0.1748	0.7399	0.4856	0.039*	
H22B	0.1115	0.9055	0.4608	0.039*	
C23	0.20205 (16)	0.93742 (18)	0.53094 (5)	0.0318 (3)	
H23A	0.1069	0.9195	0.5429	0.048*	
H23B	0.2192	1.0546	0.5281	0.048*	
H23C	0.2842	0.8895	0.5528	0.048*	

### Atomic displacement parameters (Å^2^)

**Table:**

	*U*^11^	*U*^22^	*U*^33^	*U*^12^	*U*^13^	*U*^23^
S1	0.02665 (15)	0.02229 (15)	0.01482 (15)	0.00548 (10)	0.00284 (11)	−0.00166 (10)
S2	0.03252 (17)	0.02058 (15)	0.01754 (15)	0.00567 (11)	0.00031 (11)	−0.00097 (11)
N1	0.0222 (5)	0.0236 (5)	0.0173 (5)	0.0000 (4)	0.0039 (4)	−0.0038 (4)
N2	0.0220 (4)	0.0192 (5)	0.0162 (4)	−0.0009 (4)	0.0037 (4)	−0.0011 (4)
N3	0.0331 (6)	0.0391 (6)	0.0201 (5)	0.0026 (5)	0.0041 (4)	0.0027 (5)
C1	0.0197 (5)	0.0195 (5)	0.0159 (5)	−0.0020 (4)	0.0046 (4)	−0.0009 (4)
C2	0.0200 (5)	0.0189 (5)	0.0181 (5)	−0.0029 (4)	0.0041 (4)	−0.0002 (4)
C3	0.0211 (5)	0.0222 (6)	0.0160 (5)	−0.0025 (4)	0.0040 (4)	0.0000 (4)
C4	0.0202 (5)	0.0248 (6)	0.0164 (5)	−0.0033 (4)	0.0044 (4)	−0.0028 (5)
C5	0.0415 (7)	0.0244 (6)	0.0157 (6)	0.0082 (5)	0.0039 (5)	−0.0031 (5)
C6	0.0300 (6)	0.0202 (5)	0.0151 (5)	0.0030 (5)	0.0040 (4)	−0.0043 (4)
C7	0.0282 (6)	0.0242 (6)	0.0213 (6)	−0.0003 (5)	0.0057 (5)	−0.0051 (5)
C8	0.0350 (7)	0.0303 (7)	0.0202 (6)	0.0068 (5)	−0.0023 (5)	−0.0066 (5)
C9	0.0537 (8)	0.0286 (7)	0.0154 (6)	0.0076 (6)	0.0057 (5)	−0.0008 (5)
C10	0.0432 (7)	0.0352 (7)	0.0257 (7)	0.0001 (6)	0.0171 (6)	−0.0006 (6)
C11	0.0279 (6)	0.0328 (7)	0.0248 (6)	0.0020 (5)	0.0063 (5)	−0.0036 (5)
C12	0.0246 (5)	0.0202 (6)	0.0195 (6)	0.0065 (4)	0.0028 (4)	−0.0019 (4)
C13	0.0272 (6)	0.0258 (6)	0.0263 (6)	0.0048 (5)	0.0068 (5)	−0.0008 (5)
C14	0.0334 (7)	0.0391 (7)	0.0258 (7)	0.0138 (6)	0.0129 (5)	0.0052 (6)
C15	0.0354 (7)	0.0463 (8)	0.0184 (6)	0.0194 (6)	−0.0004 (5)	−0.0049 (6)
C16	0.0304 (6)	0.0337 (7)	0.0288 (7)	0.0072 (5)	−0.0056 (5)	−0.0087 (6)
C17	0.0270 (6)	0.0232 (6)	0.0253 (6)	0.0038 (5)	0.0011 (5)	−0.0009 (5)
C18	0.0254 (5)	0.0260 (6)	0.0174 (5)	−0.0019 (5)	0.0061 (4)	−0.0016 (5)
C19	0.0241 (6)	0.0292 (6)	0.0170 (6)	0.0001 (5)	0.0026 (4)	−0.0062 (5)
C20	0.0264 (6)	0.0231 (6)	0.0153 (5)	−0.0005 (5)	0.0044 (4)	−0.0019 (4)
C21	0.0287 (6)	0.0251 (6)	0.0133 (5)	0.0007 (5)	0.0033 (4)	−0.0005 (5)
C22	0.0375 (7)	0.0385 (8)	0.0241 (7)	−0.0092 (6)	0.0123 (5)	−0.0112 (6)
C23	0.0401 (7)	0.0361 (7)	0.0215 (6)	−0.0031 (6)	0.0125 (5)	−0.0071 (6)

### Geometric parameters (Å, °)

**Table:**

S1—C1	1.7489 (12)	C12—C17	1.3861 (18)
S1—C5	1.8279 (12)	C12—C13	1.3902 (18)
S2—C2	1.7616 (12)	C13—C14	1.3862 (19)
S2—C12	1.7766 (12)	C13—H13	0.9500
N1—C1	1.3408 (15)	C14—C15	1.389 (2)
N1—C4	1.3412 (16)	C14—H14	0.9500
N2—C2	1.3253 (15)	C15—C16	1.384 (2)
N2—C1	1.3383 (15)	C15—H15	0.9500
N3—C18	1.1508 (17)	C16—C17	1.3875 (19)
C2—C3	1.4043 (16)	C16—H16	0.9500
C3—C4	1.4008 (17)	C17—H17	0.9500
C3—C18	1.4342 (16)	C19—C20	1.5324 (16)
C4—C19	1.4993 (16)	C19—H19A	0.9900
C5—C6	1.5066 (16)	C19—H19B	0.9900
C5—H5A	0.9900	C20—C21	1.5229 (16)
C5—H5B	0.9900	C20—H20A	0.9900
C6—C7	1.3923 (17)	C20—H20B	0.9900
C6—C11	1.3935 (17)	C21—C22	1.5167 (17)
C7—C8	1.3875 (18)	C21—H21A	0.9900
C7—H7	0.9500	C21—H21B	0.9900
C8—C9	1.387 (2)	C22—C23	1.5241 (17)
C8—H8	0.9500	C22—H22A	0.9900
C9—C10	1.383 (2)	C22—H22B	0.9900
C9—H9	0.9500	C23—H23A	0.9800
C10—C11	1.3885 (19)	C23—H23B	0.9800
C10—H10	0.9500	C23—H23C	0.9800
C11—H11	0.9500		
			
C1—S1—C5	101.12 (6)	C12—C13—H13	120.3
C2—S2—C12	100.01 (5)	C13—C14—C15	119.97 (13)
C1—N1—C4	116.08 (10)	C13—C14—H14	120.0
C2—N2—C1	116.35 (10)	C15—C14—H14	120.0
N2—C1—N1	127.45 (11)	C16—C15—C14	120.17 (12)
N2—C1—S1	118.03 (8)	C16—C15—H15	119.9
N1—C1—S1	114.51 (9)	C14—C15—H15	119.9
N2—C2—C3	121.48 (11)	C15—C16—C17	120.33 (13)
N2—C2—S2	119.07 (9)	C15—C16—H16	119.8
C3—C2—S2	119.45 (9)	C17—C16—H16	119.8
C4—C3—C2	117.60 (10)	C12—C17—C16	119.20 (13)
C4—C3—C18	122.05 (11)	C12—C17—H17	120.4
C2—C3—C18	120.27 (11)	C16—C17—H17	120.4
N1—C4—C3	121.03 (11)	N3—C18—C3	179.00 (14)
N1—C4—C19	116.87 (11)	C4—C19—C20	112.38 (10)
C3—C4—C19	122.10 (11)	C4—C19—H19A	109.1
C6—C5—S1	109.64 (8)	C20—C19—H19A	109.1
C6—C5—H5A	109.7	C4—C19—H19B	109.1
S1—C5—H5A	109.7	C20—C19—H19B	109.1
C6—C5—H5B	109.7	H19A—C19—H19B	107.9
S1—C5—H5B	109.7	C21—C20—C19	111.41 (10)
H5A—C5—H5B	108.2	C21—C20—H20A	109.3
C7—C6—C11	118.81 (11)	C19—C20—H20A	109.3
C7—C6—C5	121.39 (11)	C21—C20—H20B	109.3
C11—C6—C5	119.79 (11)	C19—C20—H20B	109.3
C8—C7—C6	120.46 (12)	H20A—C20—H20B	108.0
C8—C7—H7	119.8	C22—C21—C20	113.81 (10)
C6—C7—H7	119.8	C22—C21—H21A	108.8
C9—C8—C7	120.31 (12)	C20—C21—H21A	108.8
C9—C8—H8	119.8	C22—C21—H21B	108.8
C7—C8—H8	119.8	C20—C21—H21B	108.8
C10—C9—C8	119.61 (12)	H21A—C21—H21B	107.7
C10—C9—H9	120.2	C21—C22—C23	112.67 (11)
C8—C9—H9	120.2	C21—C22—H22A	109.1
C9—C10—C11	120.23 (12)	C23—C22—H22A	109.1
C9—C10—H10	119.9	C21—C22—H22B	109.1
C11—C10—H10	119.9	C23—C22—H22B	109.1
C10—C11—C6	120.56 (12)	H22A—C22—H22B	107.8
C10—C11—H11	119.7	C22—C23—H23A	109.5
C6—C11—H11	119.7	C22—C23—H23B	109.5
C17—C12—C13	120.91 (12)	H23A—C23—H23B	109.5
C17—C12—S2	119.56 (10)	C22—C23—H23C	109.5
C13—C12—S2	119.52 (10)	H23A—C23—H23C	109.5
C14—C13—C12	119.39 (13)	H23B—C23—H23C	109.5
C14—C13—H13	120.3		
			
C2—N2—C1—N1	0.16 (17)	C11—C6—C7—C8	1.37 (19)
C2—N2—C1—S1	179.08 (8)	C5—C6—C7—C8	−177.55 (11)
C4—N1—C1—N2	−0.37 (18)	C6—C7—C8—C9	−0.2 (2)
C4—N1—C1—S1	−179.33 (8)	C7—C8—C9—C10	−0.9 (2)
C5—S1—C1—N2	2.05 (10)	C8—C9—C10—C11	0.7 (2)
C5—S1—C1—N1	−178.89 (9)	C9—C10—C11—C6	0.5 (2)
C1—N2—C2—C3	0.71 (16)	C7—C6—C11—C10	−1.52 (19)
C1—N2—C2—S2	−179.12 (8)	C5—C6—C11—C10	177.42 (12)
C12—S2—C2—N2	1.98 (10)	C2—S2—C12—C17	−100.77 (10)
C12—S2—C2—C3	−177.85 (9)	C2—S2—C12—C13	78.09 (10)
N2—C2—C3—C4	−1.29 (17)	C17—C12—C13—C14	1.61 (18)
S2—C2—C3—C4	178.54 (8)	S2—C12—C13—C14	−177.23 (9)
N2—C2—C3—C18	−178.17 (10)	C12—C13—C14—C15	−1.89 (19)
S2—C2—C3—C18	1.66 (15)	C13—C14—C15—C16	0.4 (2)
C1—N1—C4—C3	−0.27 (16)	C14—C15—C16—C17	1.4 (2)
C1—N1—C4—C19	−179.88 (10)	C13—C12—C17—C16	0.14 (18)
C2—C3—C4—N1	1.05 (17)	S2—C12—C17—C16	178.98 (10)
C18—C3—C4—N1	177.88 (11)	C15—C16—C17—C12	−1.62 (19)
C2—C3—C4—C19	−179.36 (11)	N1—C4—C19—C20	72.64 (14)
C18—C3—C4—C19	−2.54 (18)	C3—C4—C19—C20	−106.96 (13)
C1—S1—C5—C6	−161.95 (9)	C4—C19—C20—C21	174.92 (10)
S1—C5—C6—C7	−72.54 (14)	C19—C20—C21—C22	−178.46 (11)
S1—C5—C6—C11	108.55 (12)	C20—C21—C22—C23	−179.41 (12)

### Hydrogen-bond geometry (Å, °)

**Table:**

Cg1 is the centroid of the C6–C11 ring.

**Table:**

*D*—H···*A*	*D*—H	H···*A*	*D*···*A*	*D*—H···*A*
C21—H21b···Cg1^i^	0.99	3.00	3.8443 (14)	148

Symmetry codes: (i) −*x*+1/2, *y*+1/2, −*z*+1/2.
